# Embolization of Ruptured Aneurysm Arising From Basilar Artery Fenestration Using Hydrocoils

**DOI:** 10.7759/cureus.326

**Published:** 2015-09-18

**Authors:** Karuna Tamrakar, Duan Chuan Zhi

**Affiliations:** 1 Neurosurgery, College of Medical Sciences, Bharatpur; 2 Department of Interventional Neurosurgery, Zhujiang Hospital of Southern Medical University

**Keywords:** basilar artery fenestration, embolization, hydrosoft coils, ruptured aneurysm

## Abstract

Aneurysms arising from the basilar artery fenestration are considered among the rare cerebrovascular diseases. Here, we report on a 44-year-old gentleman who presented with the sudden onset of severe headache complicated by several episodes of vomiting and an altered level of consciousness. A subarachnoid hemorrhage in the interpeduncle and ambient cisterns was detected by computed tomography of the head. During left vertebral arteriography, a basilar fenestration with a ruptured aneurysm just above the proximal end of vertebrobasilar junction was identified. The aneurysm was successfully occluded by means of endovascular treatment using Hydrosoft coils. In the 15-month follow-up angiography, 100% occlusion without recurrence and recanalization was observed. Bilateral anterior inferior cerebellar arteries and both channels of the basilar artery fenestration were entirely filled in follow-up angiograms.

## Introduction

Recently, authors have reported the soft hydrocoil as an effective embolic material to provide durable and progressive angiographic occlusion [1]. Although endovascular technology is the most accepted means to treat all types of intracranial aneurysms, basilar artery fenestration aneurysm embolization remains a challenge because of the risk of involving occlusion side branches and neck recurrence. Here, we describe a case of a ruptured aneurysm of basilar artery (BA) fenestration that was successfully occluded by the soft type of volume expansile coils, preserving the fenestrated segments and side branches.

## Technical report

### Case presentation

**Figure 1:**
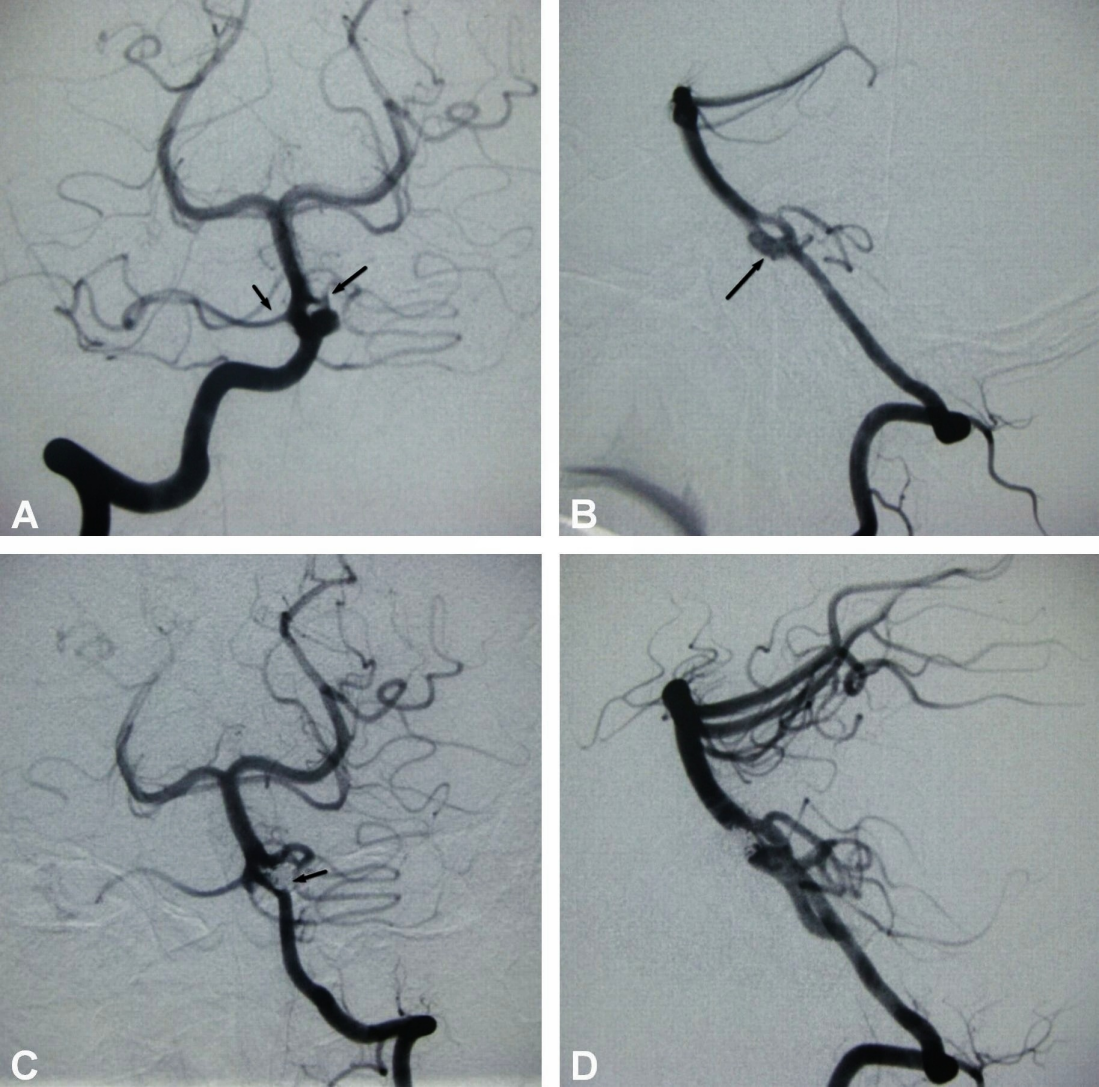
Basilar fenestration with ruptured aneurysm: pre and post-embolization results A, B: Left vertebral arteriography, anterior posterior and lateral views: Basilar fenestration is seen just above the vertebrobasilar junction with a 4 mm x 5 mm-sized ruptured aneurysm at the proximal end of the fenestration growing towards anterior- superior direction (long arrow). AICA are originating from fenestrated limbs bilaterally (arrows), C: Immediate post-embolization vertebral angiography: Near complete occlusion of an aneurysm after HydroSoft coils being positioned as finishing coils (arrow), D: 15-month follow-up angiography showing complete and stable occlusion of the aneurysm sparing the side branches.

A right-handed 44-year-old gentleman was referred to our hospital for the sudden onset of a severe headache complicated by dizziness and vomiting for several episodes. After a couple of hours of an intractable headache, he lost consciousness (Hunt & Hess Grade IV). His family members gave no history of a head injury or any bleeding disorder. The informed patient consent was signed by the family due to the inability of the patient to sign it. On neurological examination, the patient was comatose with severe nuchal rigidity. Movement of the right-sided limbs was graded as 2/5. Immediate computed tomography (CT) scan of the head showed a diffuse subarachnoid hemorrhage (SAH) in the interpeduncle and ambient cisterns extending into the ventricular system, suspicious for a ruptured intracranial aneurysm, most likely in the vertebrobasilar circulation. A four-vessel digital subtraction angiography was performed on the following day. In the selective left vertebral arteriography, a basilar fenestration was demonstrated just above the vertebrobasilar junction (Figure 1A), and a 4 mm x 5 mm-sized ruptured aneurysm was seen arising from the proximal end of the fenestration. Bilateral anterior inferior cerebellar arteries (AICA) were found to be originating from the lateral surface of both fenestrated limbs of the BA (Figure 1A).

### Endovascular intervention

The patient underwent endovascular treatment in the same setting under sedation and intravenous analgesia for peri-procedural neurologic monitoring. The procedure was performed with a biplane fluoroscopic and road mapping technique. Prowler Select Plus microcatheter (Cordis, Endovascular, Miami Lakes, FL) was navigated over the Synchro 0.014 (Boston Scientific) micro guidewire via the left vertebral artery and the tip was placed at the aneurysmal dome. Firstly, 4 mm x 10 cm and 3 mm x 7 cm-sized complex 1D Microplex 10 coils (MicroVention, Terumo, Inc.)  were deployed, framing the inner circumference of the aneurysm. The middle part of the frame was filled with an additional 3 mm x 7 cm-sized complex 1D Microplex 10 coil. Remaining bare spaces between the deployed coils and the neck of the aneurysm was then nearly packed with HydroSoft Helical 10 (MicroVention, Terumo, Inc.) coils of sizes, 2 mm x 6 cm and 2 mm x 2 cm, as finishing coils. The total length of the HydroSoft coil deployed was 10 cm. In the immediate post-embolization angiography, the aneurysm was near completely occluded and bilaterally AICA and fenestrated limbs of the BA were exclusively visualized (Figure 1C). No coil length was found herniated into the parent vessel or into the side branches. The post-embolization period was uneventful. He was then transferred to the neurosurgical intensive care unit. Spinal fluid drainage was kept into the lumbar subarachnoid space and was continued until the CSF color changed to non-hemorrhagic. He gradually recovered when the SAH gradually resolved in subsequent CT scans and was discharged with a Glasgow outcome score of 4. No event of thromboembolic complications in the distribution of the posterior fossa perforators was detected when he was clinically followed up at the three, six, and 12-month intervals post-embolization. In his 15-month follow-up angiography, 100% obliteration was detected without recurrence or recanalization and brain stem perforators were normal (Figure 1D). 

## Discussion

When bilateral longitudinal neural arteries fail to fuse completely till an embryo grows to 12 mm crown-rump length at the fifth week of gestational age, BA segmentation occurs [2]. Aneurysmal growth in the fenestration site is believed to be due to a turbulence flow mechanism as detected in branching sites of the circle of Willis. Until recently, intracranial aneurysms have been treated via endovascular therapy with successful results. Endovascular therapy of side wall aneurysms is relatively easier; however, bifurcation lesions are difficult to treat effectively. Moreover, clinical and anatomical factors like SAH, posteriorly located aneurysms, and terminus types of aneurysms, like in our case, are commonly associated with recurrence and recanalization [3-4]. Despite a narrow neck, the chance of coil migration into the parent vessel is another grave complication due to the hemodynamic effect in branching vessels, which may lead to inadvertent thromboembolic complications. Hence, various stent-assisted endovascular techniques are generally crucial because of the extreme fear of coil migration into the parent vessel, particularly when the aneurysm dome is positioned in an upward direction (Figure 1B).

Most of the vertebrobasilar fenestration aneurysms reported previously were embolized using Guglielmi detachable coils [5-8]. However, recanalization has been reported using bare platinum coils with stent-assisted coil embolization by initial angiography [9-10]. Bare platinum coils as a single embolic mass are not resilient enough to withstand the hemodynamic forces in branching aneurysms. The main concern with the use of bare platinum coils is a relatively higher rate of recanalization and incomplete occlusion [3, 11].

In an attempt to deploy the fourth Microplex-10 platinum coil into the aneurysmal sac, it prolapsed into the BA several times. If we continued packing the platinum coil densely, it would have migrated into the parent artery or into the side branches. We, therefore, decided to coil loosely with softer coils and consequently proceeded with HydroSoft coils to fill up the remaining bare spaces, including the aneurysmal neck. Near complete embolization was detected in the initial angiogram; the 15-month follow-up angiogram showed 100% occlusion, sparing all the visible perforators and fenestrated limbs of the BA. To the best of our knowledge, this is the first case reported on a ruptured basilar artery fenestration aneurysm successfully treated using HydroSoft coils.

The HydroSoft coil is a type of micro hydrolysis detachable coil, which is covered with hydrogel (hydrophilic acrylic polymer) as an inner core and platinum as an outer layer. It has an ability to expand 2-11 times more than the ordinary platinum coil of exact length due to the disentanglement of polymer chains in its surface when it comes in contact with blood [12]. Inadequately dense packing volume usually does not lead to incomplete occlusion when the bare spaces between bunches of bare platinum coils are gradually filled by volume expansile coils. However, the outer hydrogel-coated Hydrocoils are even stiffer than ordinary platinum coils, which require longer deploying time and stable positioning of the microcatheter tip. Therefore, the Hydrocoil expands earlier than the HydroSoft coil, which made it restricted to detach at the aneurysmal neck and near to the parent vessel interface. Hence, the Hydrocoil cannot be used as a finishing coil and generally is used as a filling coil at the dome region. Hydrosoft coils, being softer due to the platinum coat in its outer surface, enables repositioning and retrieving of the coil as well as the microcatheter tip during the procedure. Furthermore, the HydroSoft coil does not require pre-hydration for softening as the Hydrocoil does. For this reason, HydroSoft coils are basically chosen as a finishing coil to embolize over the aneurysmal neck satisfactorily. Gel expansion at the neck is expected to form thicker neointimal tissue and potentially helps to prevent aneurysm neck recurrence [1, 12].

## Conclusions

Incomplete occlusion usually leads to fatal consequences in ruptured intracranial aneurysms during or after embolization procedure. However, considering the necessity of preserving brain stem perforators arising from the fenestration and to prevent recanalization and recurrence, endovascular treatment using HydroSoft coils is a preferable means of therapy to gain good therapeutic outcome.
